# Lithotrity and Lithotomy Compared—An Analytical Examination of the Present Methods of Treating Stone in the Bladder

**Published:** 1832-07-01

**Authors:** 


LITHOTRITY AND LITHOTOMY COMPARED; BEING AN ANALYTICAL EXAMINATION OF THE PRESENT METHODS OF TREATING STONE IN THE BLADDER, WITH SUGGESTIONS FOR RENDERING LITHOTRITY APPLICABLE TO THE DISEASE IN ALMOST ALL ITS STAGES AND VARIETIES, AND REMARKS ON THE GENERAL TREATMENT OF GRAVEL AND STONE. By *Thomas King*, M.D., M.R.C.S. Surgeon to his Excellency the French Ambassador, Lecturer on Surgery, &c. &c. &c. 8vo. pp. 320—three Lithog. Plates. London, 1832.

IT is a difficult matter, and history proves it, to decide on the merits of any innovation, or any proposition running wide of established practice or doctrine. The cause of this difficulty is not obscure. Reason will enable a few men to ascertain the essential value of thoughts or of things, with little aid from experience. But there are highly gifted beings, far surpassing the mass of mankind in intellectual powers. We usually form our comparisons from experience; we know that one object is more pleasing or more profitable than another, because we have seen, or have found, or have learnt from the observations of others that it has been so. Our data are founded in the evidence of experience, and judgment for the most part consists in the soundness of the reasonings based on, and conclusions drawn from them. Thus it is that men rarely decide amiss on subjects admitting of experience, and thus it is that errors so signal have been committed where this great instrument of human knowledge was not, or could not be applied. The delusions of astronomy, the history of the Harveian doctrine of the circulation of the blood, are obvious and notorious instances of this fact.

In former times innovations were regarded with more than indifference; they were looked on with positive repugnance, with absolute horror. We need not go farther than the Galilean persecution for an illustrious example.

The cause of this is to be sought and found in the narrow circle to which education was confined, and the cramped and scholastic character of education itself. These circumstances combined to produce a certain train of thinking, in a certain set of men, the most unfavourable to the investigation of new views, or the unprejudiced examination of those which custom and antiquity had established. These causes can hardly be imagined to operate at the present day. Education is no longer scholastic, no longer limited to special classes; all men would learn all things, and if they have not hitherto succeeded, it is not from want of inclination on their own parts, or of confident promises from those who teach. So far as the influence of education extends, it may now be considered as rather tending towards hasty assent to what is plausible and seems new, than to obstinate rejection of what is alike new and true. Undoubtedly there is and always will be in the world, a conservative party, high-tory doctrinaires, inclined to risk every thing in defence of old opinions. With them, every notion to which they have been habituated is a fortress to be defended against all attacks, and the worse the wall and wider the breach the more gallant do they deem the defence to be, the more imperative on them the refusal to abandon it. This party, however, is weak and is daily growing weaker; the majority, as we have remarked, are rather disposed to embrace than to repudiate novelties, as such.

The candid and cautious reasoner finds how difficult it is to pronounce upon the merits of new proposals in the arts or sciences. Of the merits and defects of the old plan he is aware from the almost unerring lessons of experience; but what is to inform him of those of the new? Can he, should he trust the specious promises of the inventor, or rather should he not endeavour to exercise his reason, and be guided in the main by its decisions? We think that he should; but we also think that, as the surest basis for such reasoning and support of it is experience, which in such a case is necessarily wanting, no man of candour or of philosophic mind will venture to offer an uncompromising or dogmatic opinion, unless the proposition submitted to his examination is unequivocally confirmed or opposed by common and recognized principles or facts.

These remarks seem to us to be applicable to the question of lithotrity and lithotomy, and the actual state of those respective operations. When lithotrity was proposed we affirm that no surgeon could pronounce upon its merits. He had no experience of such an operation; he could have no data of sufficient accuracy to enable him to tell where it failed or where it answered. Reasoning from what he knew might enable him to form analogical opinions on the probable effects of what he did not know, but it could not do more. He was conscious that lithotomy was an old operation, built up by a series of failures and successes, adopted after many trials and abandonments of other operations either more ancient, or proposed in modern times as substitutes for it. He was also conscious that many futile efforts had been made to remove the stone by other means than the knife, by caustics, solvents, drilling, bruising, and that all ultimately failed. Yet, on the other hand, he perceived that most of these attempts, and especially the mechanical ones, had been engaged in at a comparatively early period, when mechanical resources were greatly inferior to what we now possess, and that, independent of the clumsiness of the instruments, their application was not then directed, as it might be now, by an accurate acquaintance with the chemistry of the urine and its concretions, or a just discrimination of what should or should not be attempted on the living body. These considerations, and such as these, must have influenced all judicious surgeons in withholding a positive opinion on the value of lithotrity when first proposed.

Ten years have elapsed since that epoch, and what is the state of lithotrity now? It has certainly gained ground, and every thinking man is convinced of its applicability and value. But we question if their real amount is ascertained, or if it will be ascertained till the operation is in more general use than at present. Many circumstances combine to render it difficult to determine the utility of any art, or branch of art, whilst practised by only a few individuals. Such was the case with the treatment of diseases of the eye, in the hands of the oculists. We therefore hail with pleasure the attempts of surgeons to make themselves acquainted with the practical application of lithotrity, and we are convinced that such attempts, if properly appreciated and encouraged, are certain, whatever be the immediate result, to end in ultimate benefit to the public.

Dr. King, the author of the volume before us, is calculated by his accurate anatomical knowledge, as well as by the opportunities which he enjoyed, and situation which he held at the Hôtel Dieu, to embark in such an undertaking. We shall put our readers in possession of the chief facts elicited by his researches, experiments, and experience.

The first twenty-seven pages of the volume are occupied by a sketch of the urinary apparatus, and it seems to us to be correctly, and cautiously drawn up. Perhaps it displays a little too much of the French mode of thinking respecting Nature's contrivances and mechanism, and scarcely evinces enough of that old fashioned English prejudice in favour of the admirable handicraft of the CREATOR, which we are neither afraid nor ashamed of avowing. There is only one passage on which we think any comment required. Dr. King observes that,—

"In almost every case it (the urine) becomes acid soon after its expulsion, thus differing from all other animal fluids, which seem to be alkaline; whilst those of vegetables are acid." 4.

This is doubly incorrect. The urine in health is acid in the bladder, nay it is secreted acid. To prove this assertion, we have only to draw off the urine with a catheter, and, retaining the instrument in the bladder, to test the urine as it comes from the kidney. The urinary is not the only acid secretion. The perspiration is acid—free acid is frequently, if not generally, secreted by the stomach—the saliva is often decidedly acid, and probably it is always slightly so.

The next chapter or section contains a description of the bladder in the adult male subject. This anatomical description is precise and perspicuous. The following quotation is deserving of consideration.

"We now come to the relative position of the bladder. It is, however, of extreme importance that the Surgeon should first know that it is a fixed organ, not a floating one. There is one part of it as immoveably fixed as any soft parts in the body; yet, this fact seems to have escaped anatomists, or to have appeared to them either too evident or of too little consequence to demand particular notice. The bladder is fastened, at its neck, to the pelvis, nearly as strongly as tendons are to the bones into which they are inserted ; and, in this respect, it differs materially from every other organ in the abdomen. Its anterior and inferior part is fixed by those strong fibrous fasciculi described by authors as the anterior ligaments of the bladder; by the triangular ligament of the urethra, which is continuous along the membranous portion of the urethra with the capsule of the prostate gland; and, indeed, by the whole of the strong fascia of the pelvis. I do not mean to affirm that these parts are as strong as tendons, or ligaments : but they certainly yield as little without laceration or injury; and as this is one of the most important facts connected with the treatment of stone, I particularly solicit attention to it.

The anterior region of the bladder corresponds to the symphysis pubis ; to the pubic ligament, from which it is separated by its own anterior ligament and cellular tissue ; and, in a small extent, to the triangular ligament of the urethra. Above the symphysis, this region corresponds, opposite the linea alba, to the fascia transversalis of Sir A. Cooper ; but whenever the bladder rises fairly an inch and a half above the pubis, it is in contact with the peritonæum lining the wall of the abdomen, in addition to its own peritonæal covering. In other words, the shining surface of the peritonæal covering of the bladder is in contact with the same surface of the peritonæum lining the muscles of the abdomen ; so that an instrument, to penetrate this part of the organ, must traverse the peritonæum twice.

I am induced to lay claim to the discovery of this fact; for all the authors I have read state, that when the bladder is distended so as to rise above the pubis, it passes to a considerable extent between the peritonæum and the abdominal muscles, or rather between it and the fascia transversalis. I was led to this discovery, from having seen the peritonæum wounded in the high operation for stone, by the best operators ; which I could not explain, till I observed, on investigating the subject, that when the bladder is distended by insufflation, it rises in the proper cavity of the peritonæum. I do not pretend that a small part of the bladder, thus distended, may not be uncovered by the peritonæum above the pubis ; but I positively assert that this organ (and in old persons more especially) expands in the abdomen, in some such manner as the uterus does in gestation, by a gradual yielding of its peritonæal as well as of its other coats ; and not by detaching the peritonæum, as it has been hitherto supposed, from the abdominal parietes. It is remarkable that an acquaintance with the nature of serous membranes did not lead, *à priori*, to a knowledge of this fact. Why should not the serous membrane of the bladder yield as much as its mucous and muscular coats ; when it is well known, that the susceptibility to yield to distension, is one of the characteristic properties of serous membranes ? Indeed, they yield more promptly than other membranes, as we see in hydarthrus, hernia, ascites, and in a multitude of other circumstances." 33.

We believe that Dr. King is perfectly correct in this description. We have seen the peritonæum reflected on itself by the distended bladder in the dead body. We remember a case in which this occurred to a very remarkable degree. A female was under the care of a physician for an abdominal tumour, supposed to be an enlarged ovarium, or a gravid uterus, or something else not very obvious. Many consultations were held on the subject, but the only point on which all appeared unanimous, was in steering clear of an operation. The woman died, and was examined. On reflecting the abdominal integuments, a tumour was seen, of considerable size, lying loosely, as it seemed, in the cavity of the abdomen. The physician chuckled —" I told you the lady was enceinte," said he : but the Doctor was premature—it was a bladder so enormously distended, that it held, at the least, half a gallon of urine. On emptying the viscus, a malignant polypus was found in the uterus, growing from its fundus. We mention this case, because the loaded bladder lay loosely in the peritonæum, like another abdominal organ, and the instrument which punctured it would have penetrated two folds of the serous membrane. At the same time we must not forget that, when the urinary bladder is distended, there certainly is a cellular interspace of some dimensions between the peritonæum, reflected from the abdominal parietes to the organ, and the upper margin of the symphysis pubis.

Dr. King very properly points out the laxity of expression respecting the "neck of the bladder," and "prostatic part of the urethra." In point of fact, these are one and the same, for the prostate forms the whole floor, and a portion of the sides of the neck, and if this be deemed a part of the urethra, then the bladder can have no neck whatever. The urethra should be considered as commencing at the anterior extremity of the prostate.

The following dimensions of the apertures of the pelvis, in reference to lithotomy, are deserving of attention. We seldom see so much accuracy displayed in anatomical description as is done by Dr. King.

"The margin of the inferior aperture of the pelvis is formed superiorly and anteriorly by the symphysis pubis; laterally, by the branches of the ossa pubis and of the ischia; behind, by the summit of the coccyx; and on the sides, posteriorly, by the sacro-sciatic ligaments and ischiatic tuberosities. The plane of the aperture is inclined forwards, as its axis indicates.

The best mode of measuring the opening, and of appreciating its form and direction is to remove the bladder, rectum, and all the soft parts of the perinæum and pelvis, except the obturators and pyriformes muscles, and their fasciæ. It will then be seen, that the distance from the bottom of the symphysis pubis to the tip of the coccyx, is about four inches and an eighth. The greatest transverse diameter is that taken from the back part of one tuber ischii, where the larger sacro-sciatic ligament is attached, to the same point of the opposite side; it passes immediately behind the anus and measures three inches.*

The extent of a line drawn across, along the inferior edge of the pubic ligament, from one side of the pubic arch to the other, is one inch and an eighth; lower down, opposite the posterior margin of the prostate, (and by opposite, I mean in a line intersecting another drawn parallel to the axis of the outlet, from this margin forwards,) the distance across, which has been tremendously overrated, is only one inch and three quarters. Finally, the extent of a line drawn from one ramus ischii to the other immediately in front of the anus, is two inches and a half. This last line circumscribes what is generally called the perinæum; all that is posterior to it belonging to the region of the anus.

The length of one side of the pubic arch, taken from the back part of the tuber ischii to the bottom of the symphysis pubis, is about three inches and a half.

We have given the average admeasurement, but these dimensions vary much more in the male than in the female pelvis; and almost always, what is lost in one diameter is gained in another; if the distance is augmented from the symphysis pubis to the coccyx, the transverse diameter will be found proportionably diminished; and when the latter is excessive, the antero-posterior diameter is lessened."—77.

---

* "The direction in which a stone should be extracted by the pelvic outlet is indicated by a line drawn from the incision in the prostate, towards the part of the pelvis where this diameter is taken; but, as the prostate is turned forwards and near the bones, the extraneous body and blades of the forceps must first be made to revolve on an axis formed at the posterior extremity of the incision in this gland."

Here we must take leave of the anatomy of the pelvis, though we cannot do so without bestowing a well-earned compliment on Dr. King, for the care with which he appears to have studied, and the precision with which he certainly has described it. Dr. K. next proceeds to the consideration of lithotomy in the perinæum. We shall select such passages as seem to convey new views, or contain important criticisms on old methods. Our readers are aware that many surgeons employ the bistoire cachée of Frere Cosme; indeed, the most dexterous lithotomist whom we know most commonly uses it. There may be objections, in point of principle, to the instrument, but we cannot avoid thinking it a very convenient one. However, what we wish to advert to at present, is Dr. King's alteration of this *lithotome caché*.

"I shall first explain what alterations I have made in the *lithotome caché*, and then briefly describe the operation as I prefer performing it. The original instrument consists of a sheathed knife four inches long, curved so as to be convex on its cutting edge; and of a handle with a spring for unsheathing and sheathing the knife, which passes from and into the sheath edgewise. The sheathed knife which I use, opens in like manner; but it is straight, wider than the original one, cutting in its whole extent, and only two inches long in the blade. Where the blade joins the handle, there is a sort of stop or hilt to prevent this part of the instrument going beyond the prostate; the handle is thin and six inches in length." 98.

Passing over the steps of the operation, as performed by Dr. King, in which there is more that is judicious than new, and merely noticing his antipathy to the operation by the gorget, which he denominates "stabbing in the dark," we pause at a subject of the utmost consequence—the incision of the prostate. On this point there is much discrepancy of opinion, and probably, therefore, much error. Mr. Brodie, in his lectures on calculous diseases, which were published in this Journal, particularly cautions the surgeon against carrying his incision through the prostate, so as to injure its capsule. If the latter be torn, there is nothing to protect the loose texture of the bladder from laceration, the cellular tissue in the neighbourhood is extensively injured, and the risk of infiltration of urine immeasurably augmented. Such is the doctrine of Mr. Brodie, deduced from no inconsiderable experience. It will now be shewn that Dr. King maintains opinions in all respects similar, nay, identical.

"Of the patients who submit to the lateral operation, one in seven or eight dies; and, in almost all those cases which have a fatal issue, death is produced either by the force used to extract the calculus, or by too extensive an incision in the prostate. If the incision is small in comparison with the stone, death will follow from the violence done to the bladder and surrounding parts, in the extraction; and if the incision is made sufficiently extensive to admit of the fair extraction of a stone one inch and a half in each of its two lesser diameters, death will follow from infiltration of urine.

In criticising the lateral operation, the first thing to be attended to is, then, the volume of the stone; success or failure depends upon it. If the foreign body never exceeded three inches in its lesser circumference, so that the incision in the prostate might be limited to three quarters of an inch or a few lines more, the operation, when well performed, would seldom or never be followed by fatal consequences. When it measures four inches and a half in its lesser circumference, or that the sum of its two lesser diameters amounts to three inches, the patient may recover, but the chances are very much against him; and when it exceeds this volume, death is almost sure to be the result of the operation.

The Surgeon is in this dilemma,—he must either use force, or make a long incision; the former lacerates the prostate and cellular tissue, bruizes the bladder and stretches its membranes, and shocks the nervous system: the latter prepares the way for infiltration of urine: both are fatal nearly to the same degree." 105.

This is a subject deserving, nay, demanding, the most anxious and careful consideration of lithotomists. The largest transverse diameter of the pelvic outlet is three inches; opposite the prostate it is only one inch and three quarters, of which one inch and a half is all that can be obtained by moderate pressure.

"But, the great obstacle to the extraction of a stone measuring an inch and a half in its lesser diameters is the situation of the prostate. The calculus must pass between the inner surface of the rami of the ossa pubis, and the posterior boundary of the incision in this gland; and, as the distance between these, when the incision in the prostate is not dangerously extensive, is only an inch and a quarter, it necessarily presses the anterior wall of the bladder against the bones, on the one hand, and tends to tear the back part of the prostate, on the other. As the prostate is firmly fixed, it cannot be drawn backwards, or pushed farther from the bones towards the cavity of the pelvis, without the ligaments yield or break; yield they may, perhaps, a quarter of an inch without laceration, and then a stone of the above size may be extracted, possibly without mortal injury.

But when the foreign body is larger, something must give way: the bones cannot yield, and if the calculus be not crushed, the prostate and bladder must be torn; and the effects of this laceration is almost certain death." 107.

We do believe that this is true, true at least in the main. Who that sees many great operations, but can generally predict too truly, which of them will be followed by disastrous consequences. The surgeon may find an excuse in unavoidable embarrassments, well-disposed spectators may allow of such, but their judgment whispers that the issue will be fatal, and very rarely indeed is that judgment false. For instances of the disastrous consequences of the violence in lithotomy, we may refer our readers to our review of Mr. Fletcher's Medico-Chirurgical Notes and Illustrations. The following expressions of Dr. King are strong, yet not more so than the circumstances warrant.

"The use of force to extract a stone is so surely followed by death, that I shudder to think how often Surgeons have recourse to it. It has so generally occurred to me, to foretel the issue of a case simply by the degree of force employed, that, if I witness it now, I do not hesitate in indicating to those near me when the operator has arrived at the point beyond which recovery is impossible. I shall shew presently, that a large opening in the prostate, made scientifically with the knife, is too dangerous a lesion to be allowed much longer to belong to surgery; but dangerous as it is, one would almost call it a safe and simple wound, when compared to the injury inflicted by a surgeon, who, placing his foot against the table, employs all the strength he possesses, to stretch and tear the bladder, lacerate its connecting tissues, bruise the surrounding parts, and shake, mortally shake, the whole nervous system! This anti-physiological process, this absurd and horrid practice, is so cruel, so fatal and yet so common, that there is nothing in which, as a surgeon, a man might more justly pride himself, than having contributed to abolish it." 111.

Laceration may prove fatal—first, from the immediate shock to the system. A blow on the epigastrium will produce death; contusion of any of the viscera may do the same, and if not immediately, almost invariably at a subsequent period. Why then should we feel surprised that tugging at the bladder and lacerating its coats, with its many tissues, its organic and animal sympathies, its large hypogastric plexus, should likewise prove a cause of speedy destruction? Secondly, contusion and laceration of the bladder and contiguous cellular membrane may occasion diffuse cellular inflammation, with its sequelæ, abscesses or sloughing, or depôts in other parts. Thirdly, the same conditions may ensue from infiltration of urine. Such are the effects, not hypothetical, but real, melancholy, practical effects of laceration. Those of too free an incision, are—infiltration of urine, and, we believe, diffuse inflammation of the cellular tissue independent of urinary effusion. Besides these, we are inclined to conceive, from cases which we have witnessed, that a circumscribed abscess in the deep cellular membrane of the pelvis is another consequence of too free an incision, though it may probably arise without it. The observations of our author on this head are so sensible, so consistent with the conclusions of practical surgeons that we feel regret at being unable, from want of space, to give insertion to them. We refer our readers to the work itself, and this we do more readily, as we have pointed out in a few words the real dangers, the roads and shoals in the way of the lithotomist.

"It would be difficult to say geometrically how long the incision may be made without danger; and I certainly have not the presumption to draw precisely the line, on one side of which is safety, and on the other death; but, I am convinced from meditation on the anatomical facts connected with this subject, and from the painful experience of seeing many die, that the danger of a wound in the prostate is in the direct ratio of its extent, and that an incision necessary for the extraction of a stone measuring an inch and a half in its two lesser diameters, puts life in imminent peril.

Of the two evils—the use of force, or a long incision—there can be no doubt which is the minor; since the latter subjects the patient to only one serious danger, whilst by the former he is exposed to many fatal consequences." 119.

The fact is that no incision into the bladder, be it ever so inconsiderable, is free from the risk of cellular inflammation. Wherever there is loose cellular tissue, the most trifling wound of it will, under circumstances which we cannot well determine, and in some individuals, give rise to that inflammatory effusion which runs like wild-fire through the tissue, killing it, or occasioning secondary inflammations and deposites elsewhere. Yet this danger is incalculably increased by contusion, or too extensive incision. Several highly interesting cases are detailed by Dr. King: we shall notice two or three.

*Case* 1. (3.) "*Protot François*, aged 22 years, of a good constitution and sanguiferous temperament, had been subject to derangement in the urinary apparatus for fifteen years, and had frequently passed small calculi by the urethra. On the 15th of April, 1822, he entered the Hotel-Dieu, complaining of the usual symptoms of stone in the bladder; he suffered intensely in voiding his urine, which contained no blood, but a great deal of mucus. A spare diet and baths were ordered. On the 12th the patient was sounded and the stone recognised. On the 15th, the operation was performed according to the plan of Frére Cosme or *(méthode lateralisée.)* Incisions were first made through the soft parts of the perinæum up to the membranous portion of the urethra; this canal was then opened to admit the sheathed knife, which was passed into the bladder, opened, and then withdrawn unsheathed, so as to divide the prostate to the extent of a little more than an inch. The stone which appeared to be very large, was extracted with great difficulty; after several attempts to remove it, which lasted eight or ten minutes, its external layers at length gave way and were crushed; it was then withdrawn without further violence. As several of the fragments escaped from the forceps, a few minutes were occupied in clearing and washing out the bladder.

The patient was carefully put to bed, and an antispasmodic *tisane* was ordered. He passed the day tolerably well, although he seemed much depressed; towards evening some uneasiness was felt at the lower part of the abdomen; it seemed however, to be occasioned by the passage of gaz in the intestiues. On the 17th, there came on vomiting with increasing uneasiness in the abdomen. Forty leeches were applied to the abdomen, and diluent drinks given plentifully.

There was some abatement in the symptoms in the night, but no distinct remission. On the 18th, twenty more leeches were applied to the abdomen, and twenty to the perinæum. The patient was also placed in a bath. On the 19th the vomiting had disappeared, but the features were much changed; the pulse was wiry, the skin hot and dry, the tongue somewhat parched. More leeches, purgatives, baths and fomentations were had recourse to, but without effect: the patient died on the 21st.

*Necropsy.* The parts in the vicinity of the wound, were macerated in a brown purulent fluid. The whole of the cellular tissue of the pelvis was infiltrated with thick pus; this infiltration extended into the lumbar regions and filled the iliac fossæ.

The peritonæum presented, to the extent corresponding to the suppuration beneath it, abundant pseudo-membranous productions uniting the small intestines to one another and to the bladder. The other parts of the body were healthy; the tissue of the kidneys seemed, however, to contain a little more black blood than usual." 127.

Here it is obvious that the mischief was primarily and essentially in the cellular tissue, and that the peritonæum was secondarily affected. The period at which the symptoms supervened, and their character, are deserving of attention.

"Some time ago, Baron Dupuytren, deeply impressed with the difficulty and danger of extracting a large stone, and imagining that they might be partially obviated by an incision occupying the whole breadth of the perinæum, revived and improved the operation of Celsus. In this method the prostate is divided by a semilunar incision, or almost horizontally, on a level with its junction with the urethra; therefore, the incision extends very near, if it does not interest the margin of the gland, where its capsule splits and becomes continuous with the different layers of the fascia of the pelvis. One of the evils belonging to this plan is, of course, the facility with which the urine makes its way between these layers; and as the consequences are precisely those which are observed when, in the lateral operation, the incision is made too horizontally, I shall insert one or two cases to illustrate this point, and farther to exemplify what has been stated generally respecting the effects of lithotomy when the stone is large. First, however, it may be right to remark here, that a still greater objection to the horizontal method is the impossibility of withdrawing the stone in the direction we have so much insisted upon, as the only one offering any chance of safety." 129.

Two cases in which the Baron performed this operation are related by Dr. King. We shall take the second, not only because it is the shortest, but because it comprises several circumstances worthy of attention.

*Case* 2 (5.) Ternisien Victor, æt. 22, admitted into the Hôtel Dieu, March 12th, 1825.

"He declared, that from his infancy he had experienced pain and difficulty in voiding his urine, although, during certain intervals, the symptoms had been intermittent. He had been sounded in 1815, when a stone was recognized in the bladder: but as the symptoms were not very urgent, no curative means were then employed.

On the 12th of March, the patient's sufferings were truly distressing; the most acute pains returned at very short intervals; the fæces were involuntarily excreted by the violent contraction of the abdominal and perinæal muscles, which took place to expel the urine whenever the fluid was accumulated in the quantity of two or three ounces; and all the usual symptoms of stone existed, to an intense degree. Being of rather a plethoric habit, he was prepared by purgatives and a spare and regular diet, till the 29th, when the operation was performed by the horizontal method.

The stone which, as was afterwards seen, was equal in size to a large walnut and of a globular form, could not be extracted without considerable force exercised amidst the patient's shrieks, and the violent convulsive contraction of his abdominal muscles.

As little blood had been lost during the operation, the patient was bled shortly after it: a large, soft, hot poultice was applied to the abdomen and an antispasmodic mixture administered. Notwithstanding these means, he passed a restless night, and in the morning of the 30th another bleeding was performed. After this he seemed better, but on the 2nd of April, he complained of uneasiness in the gluteal region: his pulse was frequent, the skin hot. April 3rd, fifteen leeches were applied to the painful part. April 5th. Shivering and increase of fever, with great restlessness. (Seven leeches; Mixture containing an ounce of the *Syrup. Papaver.)*

The following days, the same symptoms prevailed, although the urine flowed freely from the wound and leeches were applied in abundance.

The 10th instant. It was ascertained that pus had formed in the cellular tissue of the left gluteal region; and a large opening was made which gave issue to a great quantity of it, of a fetid nature, and mixed with gas. The countenance was anxious and dejected, the pulse soft but rapid, the tongue white, the skin covered with copious perspiration. A little soup was now allowed. The 11th and 12th instant. The symptoms became alarming; the small pulse, dry tongue and general depression indicated a fatal issue. A little claret was administered, but on the 13th the patient expired.

*Post-mortem Examination, Thirty-six hours after Death.* The skin of the whole of the posterior region of the trunk had a livid aspect, and under it, communicating with an opening in the left gluteal region, the cellular tissue was every where infiltrated by a layer of thick, concrete, yellowish pus. The latissimus dorsi and glutæus maximus of the left side were equally drenched in pus, which extended under them as far as the pyriformis, but no farther. The kidneys were rather pale, but healthy; the right and its ureter were a little larger than those of the left side.

The bladder was contracted and empty; its mucous membrane had rather a brown aspect, and appeared somewhat thickened. Its prostatic portion presented a horizontal opening, the lips of which appeared evidently contused. The soft parts of the perinæum were divided by a semilunar incision half an inch before the anterior wall of the rectum. Some of the lymphatic glands in the pelvis were in a state of suppuration; there was also some pus in the cellular tissue between the muscles of the upper part of the right thigh.

The brain and its membranes were in the state usually observed after death by similar causes; there was a good deal of serum, infiltrated as it were, in the substance of the pia mater. The organs of circulation and digestion were healthy.

The anterior border of the right lung presented a circumscribed abscess one inch in diameter; this appeared to me to be a true vomica. In no part of the body could a tubercle be found." 141.

In cases related in the foregoing manner, abbreviation, at least useful abbreviation, is impossible. They are already abbreviated to the utmost degree, consistent with the preservation of features of importance. The preceding is an instance of the purulent deposites in the cellular membrane and in the lung, following the operation of lithotomy. One point appears to merit notice. M. Dupuytren performed the Celsian operation, or a modification of it, that is, he made a semilunar incision between the rectum and urethra, in order to gain space for the extraction of the stone. Now we are told that the latter was equal in size to a large walnut, and that considerable force was exerted in effecting its extraction. We do not hesitate to say, that a stone of this bulk could have been extracted without the employment of more violence by the lateral operation. It appears to us that it is not in the external incision, or in the wound between the integuments and the bladder, that space is required. It is the opening into the bladder itself that is insufficient for the safe removal of a large stone. It may be said that, by the Celsian operation, we divide both sides of the prostate, and so gain room. The fact is, that both sides of the prostate may be divided, independently of the Celsian operation, by a broad-shouldered, double-edged knife or gorget, with a beak inclined at an angle to the plane surface of the blade. By the Celsian operation we are liable to wound both ejaculatory ducts; nay, it is not impossible that the urethra might be severed from the bladder. These are objections; and the foregoing case is sufficient, of itself, to prove that the freedom with which a large stone can be removed by it is ideal.

The next subject adverted to by Dr. King is hæmorrhage, an important one. This is usually furnished by the perinæal artery, or the artery of the bulb, occasionally, as in the case of the late Mr. Shaw, by irregular branches which no knowledge can anticipate—no dexterity avoid. Dr. King observes, and we think well, that profuse and dangerous hæmorrhage may be furnished by small deep-seated vessels about the neck of the bladder. There is a plexus of veins in this situation, and we have good reason for thinking that, in advanced age, when the vital contractility of the bloodvessels is diminished, and the natural means of arresting hæmorrhage inefficient, bleeding to a dangerous amount may occur from this source. We also agree with Dr. King that the best, because the least injurious, means of stopping this bleeding, is pressure by the finger of an assistant. This is irksome enough, God knows; but the life of the patient is at stake.

"At the Hotel Dieu, plugging to arrest hemorrhage after perinæal lithotomy, is performed thus:—a kind of linen purse, five or six inches in length, which the French call *shemise*, a straight metallic tube or catheter not quite so long, of three eighths of an inch diameter, and a certain quantity of lint for stuffing *(bourdonnets de charpie)* are necessary for the purpose. The purse is fixed tight round the canula in a notch at about three quarters of an inch from that end of it which is to be passed into the bladder, and open at the opposite extremity. By this contrivance the canula is placed in the centre of the bag, and protrudes through it at one end. The Surgeon, after anointing the exterior of the linen with cerate, passes the canula with the purse drawn close upon it through the wound into the bladder, his left index serving as a conductor; he then introduces the bullets of lint into the bag, one by one, with a common dressing-forceps, pressing them down with sufficient force to stop the bleeding. Pressure is thus exercised between the lips of the wound, in their whole extent, and the canula which secures an issue to the urine, and which is fixed by means of two rings it presents to a T bandage adjusted to the patient." 145.

Hæmorrhage after lithotomy is a great evil on several accounts. Independent of the immediate risk from loss of blood, and subsequent exhaustion or reaction, we believe that the pressure and the plugging of the wound increase in a very material degree the hazard of the formation of matter or of diffuse inflammation in the cellular tissue of the pelvis. It is certain that in two instances which we have lately seen, of abscess in the cellular texture of the pelvis after this operation, hæmorrhage occurred, and required long and tedious pressure to restrain it, much exposure of the parts and much disturbance of them. A fatal case of hæmorrhage is related by Dr. King. The symptoms in that case are so perfectly analogous to those which we have witnessed in cases of internal suppuration, that we cannot but suspect the existence of such, although Dr. King declares that none was discovered on the examination after death.

### Lithotomy by the Rectum.

We know how this operation has been lauded in modern times. Bellinghieri, we think, in Italy, has been said to have met with we know not what success, but something almost surpassing belief. M. Sanson, at the Hôtel Dieu, has also been said to be very successful. It is fortunate that Dr. King has had an opportunity of seeing many of M. Sanson's and M. Breschet's cases, and can therefore tell us what he saw. We shall find that his remarks are not calculated to encourage English surgeons to adopt this operation.

"Recto-vesical fistula and infiltration of urine render it dangerous, and the former attends it in so consequent a manner, that it is as disastrous, if not so immediately dangerous, as the lateral method. Those who have watched cases in which this plan was adopted must allow that it leaves a fistula as an almost certain consequence of the operation. The incision will not close; it appears that its lower part constituted by the anterior wall of the rectum, is kept open by the passage of the fæces and irregular motions of the sphincter, and its upper by the flowing of the urine; there is no support for granulations: the cicatrix has nothing to build upon.

This result is so constant, that the recto-vesical section must be viewed rather as the substitution of one disease for another, than as the means of restoring health; and it then becomes a question whether the one substituted, is of such inconsiderable inconvenience as to warrant its introduction into practice? If this kind of fistula only amounted to an inconvenient and temporary deformity, the project might be entertained; but, unfortunately it is of a very serious nature, and depending as it does entirely upon the physical disposition of the parts concerned, is almost always beyond the reach of art. In general it is not immediately destructive, but it seldom persists long without impairing even the most robust and vigorous constitution. The continual contact of the urine produces inflammation in the mucous membrane, which, as it creeps along the intestine, occasions great disturbance; giving rise to diarrhœa, which cannot be subdued, since the cause cannot be removed, and which, although the patient may resist it for months, or even years, finally exhausts him, either by its own intensity, or by provoking disease in some organ whose integrity is necessary to life. I should think that one half of these cases of fistula terminate fatally. In a very small proportion of them, where the patients happen to be placed under the influence of the most propitious circumstances, a cure may be obtained; but by far the greater portion of those whose general health remains unimpaired by the local injury, must submit to a deformity, which renders life almost unendurable. I have seen, at least, five or six operations by this method, and in only one case was the cicatrisation of the wound obtained, although they were performed with consummate skill, and great care was taken to make only a mere notch in the rectum." 160.

Infiltration of urine is perhaps less likely to follow this than the lateral operation. The ejaculatory ducts may be wounded. In the only instance in which we have seen the recto-vesical operation performed, the patient died of abscess in the pelvis. Dr. King relates a case in which the operation was performed by M. Sanson. The patient died of suppuration and sloughing in the cellular membrane, extending into the peritoneal cavity.

### High Operation, or Lithotomy above the Pubes.

As Dr. King very justly observes, this operation is not only more liable than the lateral operation to be succeeded by infiltration of urine into the cellular tissue, but also by peritonitis, the two chief dangers of the latter. When we add that the operation is one of extreme difficulty, we pronounce its condemnation in the most emphatic terms. A very large stone has been supposed to be its only justification, yet we venture to say that if a calculus is too large to admit of extraction by any operation but that above the pubes, it were better not extracted at all. The patient must almost surely die.

### Lithotrity.

The following enumeration of the gentlemen who have invented lithotrity, for really there appears to have been half-a-dozen, will prove what we have frequently asserted, that in medicine, as in the arts, discovery is always gradual. The man who first strikes out an idea is seldom if ever successful in its practical application.

"Lithotrity consists in the reduction of calculi to powder, or fragments sufficiently small to pass through the urethra, by means of instruments introduced down this canal into the bladder. To accomplish this or some very similar object, instruments have been invented and recommended by Messrs. Elderton, Amussat, Leroy d'Etiolles, and Heurteloup. These gentlemen were preceded by Mr. Gruithuisen in the invention of an instrument for drilling a hole in calculi, but with a view, it is believed, of dissolving them by some chemical agents; and it is well worthy of remark, that long before their praiseworthy attempts, two non-medical persons, a monk of Citeaux and Col. Martin, not only devised but actually cured themselves of stone by means somewhat similar to those now adopted in Lithotrity. The idea of breaking and pulverizing a stone in the bladder seems first to have originated with the monk, but it is not likely Col. Martin borrowed it from him, since he employed different means. I think there is no probability of plagiarism, either on his part or that of the practitioners we have just named. It would be a difficult task to assign to each of these the degree of merit due to him; suffice it to say, they have all well deserved of mankind. Mr. Gruithuisen, who published in 1813, appears to have been the first to devise any thing like scientific or methodical means for acting upon a stone in the bladder by trituration. In 1819, our countryman, Mr. Elderton,* conceived the idea of Lithotrity, and invented ingenious instruments for the operation; but here he stopped. Mons. Leroy had the same idea in 1822; he constructed and made known instruments which are now in use. About the same time, Mons. Civiale succeeded in the same object; and to him was reserved the good fortune and high privilege of first employing Lithotrity with method and success upon the living subject: he is still a very successful operator. Baron Heurteloup is now quite as successful and scientific a lithotritist, and to him the world is indebted for the invention of instruments more ingenious, and in many respects, more perfect than those known before his valuable labours. The merit of laying down the principles of Lithotrity more comprehensively and more perfectly than any other person, belongs also to him; and he shares likewise the honour of its introduction into Great Britain, to which, with my skilful friend Mr. Costello, I hope I have endeavoured to contribute." 183.

The instruments most in use at present are, the three-branched forceps of Messrs Leroy and Civiale, and the four-branched forceps, the assisting forceps, and the shell-breaker of Baron Heurteloup. We lately gave an extended review of the able Baron's work, and, as many descriptions of the process of lithotrity and of the instruments employed are before the public, we shall refrain from touching on these topics again. Our object is rather to point out the comparative advantages of lithotrity and lithotomy, and to put our readers in possession of the sentiments of Dr. King, which appear to us both impartial and judicious, on this important subject. It seems to be generally acknowledged that, in the present state of science, lithotrity is scarcely applicable when the foreign body exceeds an inch and a half in two of its diameters. Thus large stones must still be left for the lithotomist; that is, the worst and most fatal cases must continue to be confided to him. Information, however, is spreading both in the profession and out of it, and we entertain no doubt that patients will now learn to appreciate the early symptoms of this complaint, and apply in the first instance for surgical assistance. Surgeons, again, will not only be better acquainted with the initiative of calculous disorders, with the chemical alterations of the urine, and the most scientific means of obviating them; but they are in possession of mechanical methods of destroying or removing small stones when formed —methods which, in such circumstances, are comparatively devoid of risk, and fail to inspire the horror which the prospect of lithotomy must create in the minds of unfortunate patients. The invention of Sir Astley Cooper's urethra-forceps and of lithotrity must, in this point of view, form an era in the history of surgery, and, if mitigation of human sufferings and preservation of human life deserve well of mankind, in the history of the world.

Dr. King very properly insists on the care which should be taken to prepare the patient for lithotrity, as for all great surgical operations, care which we regret to say we have not seen on too many occasions. The following are Dr. King's opinions on the three-branched instrument of M. Civiale, and the four-branched instrument of Baron Heurteloup.

"A few remarks will now be necessary upon the preference to be given to

* "To this gentleman, who resides at Northampton, we are also indebted for other inventions of great importance in surgical operations."

any particular plan of Lithotrity, or to any particular instruments, in the treatment of the principal varieties of stone. We have no hesitation in stating, that there is only one case in which the four-branched instrument ought to be preferred, namely, that where the calculus is composed chiefly of oxalate of lime, and very hard. In all other cases, which form by far the greatest number, we prefer the three-branched instrument and drill, that is, the treatment by successive perforation, which, in the present state of science, appears much more safe than any other. The instrument with which it has been proposed to destroy the stone by excavation, is much more liable to break than the three-branched forceps ; and that invented to act by concentric pulverization is open to the same objection, besides being inadequate to the purpose for which it was intended. Most assuredly we should recommend the four-branched instrument for the treatment of all large round stones, of whatever composition, if excavation, or still more, if concentric pulverization could be employed with efficacy and safety; but we contend they cannot : the excavator has not yet been, and we fear, can never be made of sufficient strength ; and even barring this difficulty, it is almost impossible to reduce a stone, in the manner proposed, to so thin a shell that it may be broken by percussion, unless it can be seized with the percussor-forceps. When an excavator shall be made so strong that there be but very little risk of its breaking, and so well as to reduce a stone to a mere shell, it will deserve to be adopted with the four-branched forceps, in preference to the drill and the three-branched instrument, wherever the calculus is large and round. But, we are of opinion that the most rational and safe plan of all for destroying calculi, would be that of concentric pulverization, were the saw sufficiently perfect to carry it into effect. Some of the strongest objections to Lithotrity would then be obviated : there would be no rough fragments left to irritate the bladder; and as the instrument would leave the surface of the stone smooth, the operation, every step of which would bring relief to the patient, might be interrupted and deferred, at any time, with impunity. Had we such a saw it would be right to use it with the four-branched forceps, in all cases where the stone is not flat, but more particularly in those in which it is voluminous. It will appear from what precedes, that we consider the four-branched forceps better adapted, than any other, to overcome the difficulty of seizing the stone, without pinching the bladder." 216.

In general, several applications of the instrument are necessary, the number varying with the size and consistence of the stone, the state of the bladder, and condition of the patient. The time between the sittings must of course vary under similar, or nearly similar circumstances. The surgeon should always be prepared to perform lithotomy, and should never be without the necessary instruments. Of the propriety of this advice, we have ourselves seen a remarkable instance. For many judicious directions and reflections respecting the operation, and matters connected with it, we refer the surgeon to Dr. King's work. After relating two interesting cases of lithotrity, he proceeds to consider, seriatim, the objections which have been advanced against it.

1. "A great deal has been said about the difficulty of seizing the stone; many considering it so great as to prevent the general adoption of the operation. The ablest Surgeons, say they, have been foiled in their attempts to lay hold of the calculus, and those who style themselves Lithotritists, nearly as often. But the answer to this is, that the difficulty, however great, (and certainly it is the greatest attending the operation) is to be overcome by patience and that dexterity which practice gives. Besides, it has been, in a great measure, obviated by Baron Heurteloup. With his bed, the stone can almost always be made to roll to the back part of the floor of the bladder, where a Surgeon who has practised on the dead subject, will seldom fail to grasp it.

2. The liability to contuse and pinch the bladder is certainly a serious objection; and it cannot be denied that these injuries are sometimes inflicted. But, although the coats of the bladder may, by chance, be caught in the forceps by an expert operator, he will discover the accident early enough to avoid doing them any fatal or serious violence. We are inclined to believe, that except on very rare occasions, he must be rather inexpert who could pinch or strike the bladder so severely as to produce contusion, laceration, or violent inflammation.

3. Another strong objection, founded upon the occurrence of the accident to some of the best Lithotritists, is, that the instruments are liable to break in the bladder. Now, admitting this may have happened once in twenty or thirty operations, when we consider that it is to be attributed to defects in the instruments, which are rendered every day more perfect and less fragile, is it too much to expect, that the time will come when such an occurrence shall be unheard of? When it does take place, the patient must generally undergo Lithotomy; but even then, he is not in a worse condition than he would have been before the invention of Lithotrity. He may possibly be in less favourable circumstances, from the irritation already produced by the Lithotritic process; in most cases, however, he will not. In a very unirritable patient, the detached piece of instrument might, perhaps, be attracted and drawn through the urethra with a magnetized rod, it might even find its way out spontaneously; but, since in most cases of this kind, Lithotomy must be performed without delay, the Surgeon should always have the requisite instruments at hand, as well as a magnet.

4. It has been stated, that, if the branches of the forceps cross each other, or become entangled in a hole of the stone, there is no possibility of withdrawing the instrument, without lacerating the urethra. This would certainly be a very embarrassing accident, and, although there is no evidence that it ever occurred, its bare possibility should induce the Surgeon to provide against it. If unable to put things right with a steel rod, he would be obliged to have recourse to an opening in the bladder, by the perinæum, through which he might disentangle the end of the instrument with his finger, or in case of this failing, cut it off with a pair of curved nippers I have had constructed for the purpose, and with which he should always be furnished. But, the best provision against such an accident, would be to have the branches of the forceps separate throughout, as recommended by Messrs. Costello and Guthrie, so that, by withdrawing the external tube, they might be removed one at a time.

5. Several eminent practitioners contend that the principle of reducing a stone in the bladder to fragments, is bad and untenable; they are of opinion, that the presence of these fragments, in so delicate an organ, is sufficient to produce dangerous inflammation; that the frequent application of the instrument required to destroy them, very often occasions the same result, and finally, that, in spite of the greatest skill and attention, one may remain unpulverized, and become the nucleus of a new calculus." 237.

Now, in answer to the last objection, or rather string of objections, Dr. King replies, that experience has proved that the fragments left after the lithotritic process do not give rise to inflammation, in the great majority of cases. Several applications of the lithotritic instruments can hardly be looked on as a serious objection, provided the risk is not materially increased, which it certainly is not. Lithotomy, it is true, is usually completed at once; but is the patient's convalescence accomplished in a day? If safety is increased by the sacrifice of time, that sacrifice should be cheerfully and unhesitatingly made. In fact, we cannot look upon this as an objection to lithotrity at all—it is merely a circumstance which diminishes its éclat, and derogates from its rapidity of cure and quantum of inconvenience. Are relapses more frequent after lithotrity than lithotomy? Probably they are so, although the cutting operation is well known not to be devoid of them. But if a relapse does occur after lithotrity, it is but, as Dr. King supposes a patient to exclaim, " only a little more grinding."

"The opponents of Lithotrity have not failed to enumerate, at full length, the cases to which it is inapplicable: those where the stone is very hard, or very voluminous, or encysted; diseases of the prostate; urethræ originally very small, or contracted from strictures, have been much dwelt upon. In considering these dispassionately, it will be found, that they do not constitute any thing like a foundation for hostility to the general adoption of the operation. In some adults, where the urethra is naturally very narrow, it may sometimes be impossible to introduce the smallest instrument; but this is very rare indeed. And, in children, Lithotomy, well performed, loses three-fourths of its dangers, differing by its lenity, nearly as much as Lithotrity does, from the same operation performed on grown persons." 244.

This is the true state of the case. Take away these cases, and you have a great number to which lithotrity is applicable, and in which it may, and in all human likelihood will, become a means of safety. With respect to encysted calculi, the remark appears absurd. We would wish to know on how many cases of encysted calculi would the surgeon operate, knowing them to be such, or in how many would an operation be required! Such frivolous objections as these are like nibbling at the operation in committee; they come from the waverers.

" After this enquiry into the principal circumstances connected with the treatment of stone, by Lithotomy and Lithotrity—in which we have attempted to place side by side, their respective dangers and advantages, by an appeal to facts, and thus to establish the degree of estimation in which each ought to be holden in practice,—our conviction is, that wherever Lithotrity can be employed, Lithotomy should never be thought of." 249.

### PROPOSALS FOR THE TREATMENT OF CALCULI OF GREAT MAGNITUDE AND DENSITY.

The surgeon whose genius could devise or dexterity accomplish means of removing large calculi from the bladder with little, or even with less than the present risk, would unquestionably deserve well of his kind. Large calculi are the opprobrium of surgery. Lithotomy for them brings nothing but danger; to them lithotrity is confessedly inapplicable. Dr. King makes some proposals for their treatment which we shall lay before our readers.

We need not repeat that a great, perhaps the greatest danger in the operation of lithotomy is a large incision. Yet, whenever the calculus is of great dimension we must either make an incision fatally extensive, or commit a laceration yet more destructive. We are on the horns of a dilemma, and, do what we will, experience has proved that lithotomy for large calculi is too generally unfortunate.

" The principle of Lithotomy—Lithotomy of the safest and best kind—is, then, to have the *incision* proportionate to the *size of the stone*. Now, what I would suggest is, that this principle be reversed, and that the *stone* be reduced to the limits of a tolerably safe incision. In Lithotomy, the rule is, to adapt the *wound* to the *calculus;* whereas, I propose, adapting the *calculus* to the *wound.* This new plan may be called perinæal Lithotrity." 252.

Such is the principle which guides Dr. King. It has been remarked that when large calculi have broken in the forceps, previously to their extraction, the patient has recovered more frequently than when no such accident has happened. The breaking of a large calculus by the lithotomist appears to the inexperienced spectator a misfortune, yet it may be questioned if it be not the reverse. Dr. King's propositions are founded on the good effects, real or reputed, of this very accident. In short he proposes to break the calculus, after making an opening, a small opening, into the bladder in the usual manner from the perinæum.

"In order to meet the objection founded upon the inconvenience of scattering the bladder with fragments, I have endeavoured to devise means for enclosing the calculus in a bag, prior to breaking it to pieces. The instrument I use for the purpose is a light forceps, having the upper blade split into two branches, which are made to open and shut, and to which the end of a bag is adjusted for receiving the calculus. When the latter is pressed in the forceps, at the same time that the branches of the upper blade are gradually expanded, it necessarily passes between them into the orifice of the bag, in which, by continuing the pressure it becomes finally enveloped.

The next object was to find the best means of breaking the stone, in the quickest possible time. I tried several instruments, some constructed on the model of the forceps of Andreas à Cruce, with long strong branches, and wedge-shaped teeth in the blades; others having a contrivance for breaking the stone with a wedge or percussor; and one like that used by Baron Heurteloup for breaking calculi by percussion. Those of the first kind are the most simple, and give the least trouble in seizing the stone, and as they are extremely powerful, few calculi will resist their action. With the others, the hardest calculi may be broken, but they do not offer the same facility for grasping them.

The operation is performed in the following manner. The patient, who should be prepared as before submitting to Lithotomy, is to be placed upon Baron Heurteloup's bed. I introduce into the bladder a slightly curved staff, which is held by an assistant, nearly on the median line, whilst I make an incision up to it through the perinæum. This incision, about an inch and a half long and extending from a central point about an inch and a half below the inferior part of the symphysis pubis towards the left side of the anus, is made in two or three strokes with a straight, pointed knife; one serving to open the urethra from the left side of the bulb backwards, as in Lithotomy. I use Blizard's knife, or a sheathed instrument, to extend the opening to the anterior part of the prostate. A small conductor is then passed through the opening into the bladder, in the groove of the staff; and the latter being withdrawn, I exchange the conductor for the forceps. Thus far the operation differs from Lithotomy, only, in as much as the incision is made just sufficiently extensive to admit the forceps. In introducing the latter, the principal part of the bag, previously oiled, is kept close to the double or upper blade, which, when the stone is grasped, is to be gradually opened. As soon as the calculus is completely enclosed, the branches are to be shut and brought near the wound, in order that the end of the bag may be made secure and detached from them. The forceps is then to be withdrawn. It is through that part of the bag hanging out of the wound, that the instrument for breaking the stone is to be introduced, guided by the finger or a conductor; and, as the patient is placed in the position required for seizing the foreign body in Lithotrity, the stone, which cannot escape from the bag, will be easily grasped." 259.

To this proposal there may be some objections, but Dr. King believes that the chief will lie against the apparatus, and this may admit of improvement almost to an indefinite extent, at least that improvement can only be limited by human ingenuity. The risk of fragments remaining in the bladder is an evil; yet supposing them not removed by the means in our power, they can leave but the necessity for future lithotrity. We must recollect that in this case we have only a choice of evils.

"The length of time required for the performance of the operation, and the necessary pain and irritation attending it, constitute another objection to perinæal Lithotrity. But, again, we ask, whether they endanger the life of the patient? What does reasoning upon analogy, what does experience teach us on this point? In Lithotomy, it is not the frequent introduction of instruments, or the time employed, that renders the operation dangerous, but the extent of the incision or the force employed to extract the stone; and, when hemorrhage is avoided, when we are secure from infiltration of urine, when the bladder has not been forced or the prostate lacerated, we may expect a successful result." 263.

In those desperate and deplorable cases, where the calculus is too large to admit of even this proposal being carried into effect, Dr. King recommends another plan. We are sorry that our exhausted space compels us to pass it over. Our own opinion is this, that in cases of such extremity, the result is but too probably unfortunate under any method of procedure, or any plan of treatment. Mankind will reap more benefit from the early discovery and removal of small stones, than from any inventions in the treatment of large ones. At the same time, we would not discourage attempts to mitigate the dangers of their extraction.

A chapter on calculus in the female bladder contains nothing of particular moment. The last division of the work consists of General Remarks on the Treatment of Calculi. In our full analysis of the able and practical lectures of Mr. Brodie on calculous disorders, their nature and treatment were so fully considered, as to render it unnecessary for us to dwell on them again. We may take the opportunity, however, of stating, that we think Dr. King's reflections indicative of judgment and reflection. But we do not quite agree with him on every point. He says, for instance, that the ammoniaco-magnesian diathesis certainly originates in a diet of too animal a nature, and, therefore, recommends the use of vegetables. That too much animal food, especially when conjoined with sedentary habits and late hours, will cause the deposition of the triple phosphate in the urine, we have the best means of knowing, having lately witnessed a well-marked instance of this description. But we doubt whether this be generally the case; indeed, we know that the triple phosphate is usually the deposition of debility—remedied by all that invigorates, augmented by all that weakens. In illustration of this principle, we refer to the excellent work of Dr. Prout. For this reason, general bleeding is seldom admissible in the triple-phosphate diathesis, and the surgeon should be cautious how he employs it.

Dr. King feels himself justified in stating, that the formation of the oxalate of lime gravel coincides with the use of coarse, indigestible food, in which animal matter does not preponderate. The causes of this diathesis have hitherto been a problem; but the deposition of the oxalate of lime in the urine appears to be connected with the formation of acid, rather than alkali, in the system, as is proved by its occasional alternation with the lithic acid in calculous concretions. Dr. King observes that in one case, in which he exhibited citric acid against oxalate of lime gravel, it succeeded perfectly. Dr. King very properly insists on dietetic rules and restrictions. It is worse than a farce to meet acid or alkali in the urine by alkaline or acid medicines, when the habits and the food which occasioned the morbid character of the secretion are allowed to be persisted in. For our own parts, we would infinitely rather throw physic into the Thames, and trust to regimen and general means only in the treatment of affections of the urinary organs, than appeal to pharmacy, to the exclusion of these most important agents. We speak advisedly; and we cannot but believe that the chemistry of the urine may possibly prove an ignis fatuus to some, and lure them from the high road of common experience and common sense.

We must now bring this article to a termination. We are not in the habit of pronouncing those oracular pieces of criticism, which seem to us of no use but to flatter or to hurt the feelings of authors. We do not wish to crucify an unfortunate writer, or exhibit him writhing under the lash of public ridicule or vituperation. We war with nothing but impudent assumption—want of principle—with doctrines or with practices calculated, in our opinion, to be mischievous to the community. If books are good, we endeavour to make the public fully acquainted with them; if indifferent, we usually pass them unnoticed. Silence is our censure—notice is our praise: by the former we lacerate no feelings—by the latter we accomplish a public good. We need say no more, then, to Dr. King than our review has told him; this is not a courtly compliment, but it is better—it is an honest one.

